# Quantum-Material Josephson Junctions: Unconventional Barriers, Emerging Functionality

**DOI:** 10.1021/acs.nanolett.6c01333

**Published:** 2026-08-26

**Authors:** Kathryn A. Pitton, Michiel P. Dubbelman, Trent M. Kyrk, Houssam El Mrabet Haje, Yaozu Tang, Roald J. H. van der Kolk, Yaroslav M. Blanter, Mazhar N Ali

**Affiliations:** † Department of Quantum Nanoscience, Faculty of Applied Sciences, 145265 Delft University of Technology, Lorentzweg 1, 2628 CJ Delft, The Netherlands; ‡ Kavli Institute of Nanoscience, Delft University of Technology, Lorentzweg 1, 2628 CJ Delft, The Netherlands

**Keywords:** superconductor, Josephson junctions, Kagome, ferroelectronic, noncollinear, quantum material

## Abstract

Josephson junctions translate quantum phase coherence into an electrical response and underpin superconducting sensors and quantum circuits. In conventional junctions, the barrier acts primarily as a passive weak link; however, when the barrier is a *quantum material* with its own internal degrees of freedom like magnetism, strong correlations, or switchable polarization, the Josephson effect becomes a sensitive probe of symmetry and many-body physics in the interlayer. Here we review progress in “quantum-material Josephson junctions” (QMJJ), focusing on three rapidly advancing barrier families: (1) magnetic barriers, where exchange, noncollinearity, and spin-active scattering enable 0−π–φ ground states, singlet–triplet conversion, and nonreciprocal transport; (2) correlated barriers, where proximity effects acquire many-body character and recent van der Waals Kagome Mott interlayers exhibit field-free Josephson diode behavior; and (3) ferroelectric and multiferroic barriers, where nonvolatile polarization provides an internal control knob and can produce superconducting memory and memristive dynamics.

## Graphical abstract

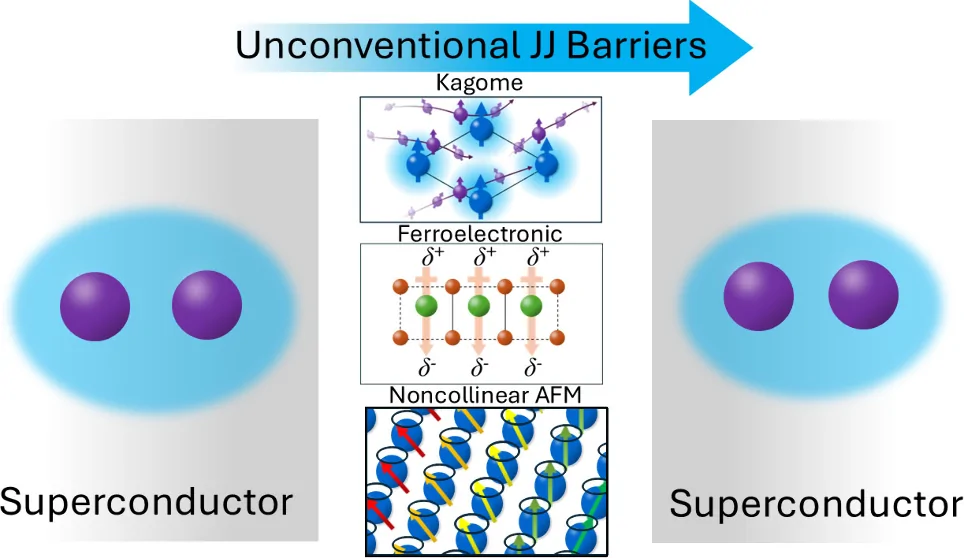

The Josephson junction (JJ)—two superconductors (SCs) coupled through a weak link—is a quintessential element of superconducting technology.^1^ The DC Josephson effect creates a dissipationless charge current due to a phase difference between two SCs, forming the basis for JJ-based sensors such as Superconducting Quantum Interference Devices (SQUIDs). JJs also exhibit the AC Josephson effect, in which a constant voltage generates an alternating supercurrent at a frequency directly proportional to the applied voltage.^2,3^ This effect provides a universal phase voltage relationship linking quantum coherence to measurable electromagnetic radiation, enabling the generation and detection of microwave radiation, underpins voltage to frequency conversion, and serves as the operational basis for superconducting standards, high-frequency detectors, and many superconducting qubit architectures. Thus, the DC and AC JJs can be engineered for use in computing, sensing, metrology, and quantum information.^4^

Although the macroscopic behavior of Josephson junctions is governed by superconducting phase coherence, the microscopic transport properties depend strongly on the nature of the barrier separating the superconductors. In JJs where the weak link between SCs is established through a nonsuperconducting barrier, the barrier properties govern the mechanism of charge transport: either tunneling in normal insulators or Andreev reflection in normal metals.^5,6^ The insulating barrier can play an active role in determining the Josephson response, like in traditional magnetic insulating barriers where the spin structure can lead to nontrivial current–phase relations (CPR) which cannot be attributed solely to generic interfacial effects. By contrast in metal barriers, there is sufficient density of states at the Fermi level for Andreev reflection to dominate, where a Cooper pair is transmitted into the metallic barrier by the injection of an electron and the formation of a hole, followed by the electron–hole pair recombination at the other superconducting interface.^6^ The microscopic transport behavior of Josephson weak links, Andreev processes, and proximity effects across a wide range of nanoscale junctions has been reviewed extensively elsewhere.^7^ While these broad barrier classes exhibit specific CPRs, the material properties that can be used to modify the behavior of JJs are far richer and more complex, with topological and ferromagnetic materials among the most studied at this time.

In this mini-review, we define “quantum-material Josephson junctions” (QMJJs) as Josephson junctions in which the barrier is not merely a passive tunneling medium but instead possesses internal quantum degrees of freedom—such as magnetic order, strong electronic correlations, or switchable polarization—that actively modify the superconducting phase coherence, current–phase relation, or transport symmetry of the junction itself. Under this framework, the defining feature is not a particular material family but rather the emergence of barrier-driven Josephson phenomena arising from collective electronic, magnetic, or ferroic behavior within the interlayer. Conventional SIS or SNS junctions, where the barrier primarily acts as a passive tunneling or metallic medium, therefore fall outside the scope of this category.

Several classes of quantum material barriers have emerged as especially promising platforms for engineering unconventional Josephson phenomena. For example, complex magnetic barrier JJs can act as an active functional element that can promote spin-triplet superconductivity, realize π-junction behavior, and operate as a spin valve for superconducting spintronic applications.^8^ Recently, altermagnets, characterized by a magnetic structure with zero net magnetization but with spin-split electronic bands arising from symmetry-protected, momentum-dependent spin polarization, are theorized to offer a platform for engineering spin-dependent superconducting transport without stray magnetic fields or strong uniform exchange-field pair-breaking effects typically associated with ferromagnetic junctions.^9^ This combination enables novel avenues for manipulating the superconducting phase, generating unconventional CPRs, and realizing spin-selective or nonreciprocal Josephson effects within a magnetically compensated system. More broadly, nonreciprocal superconducting transport and superconducting diode effects have recently emerged as a rapidly developing research direction across a wide range of superconducting systems, including noncentrosymmetric superconductors, Rashba heterostructures, and atomic-scale Josephson junctions.^10^

Correlated barrier materials, in which strong electron–electron interactions renormalize the material’s electronic properties, can directly reshape superconducting correlations through modulation of the pair amplitude, interface-pinned oscillations, and other effects, also resulting in nontraditional JJ behavior with emerging experiments in van der Waals correlated kagome interlayers reporting field-free Josephson diode behavior. Ferroelectric (FE) materials have a macroscopic, remnant, and switchable internal electric polarization, which can couple to superconducting transport when incorporated as barriers in JJs. In ferroelectric JJs, this polarization enables tunneling electroresistance, a phenomenon widely exploited in normal state ferroelectric random-access memories and field-effect transistors, offering an additional nonvolatile control parameter for superconducting tunneling.^11^ Finally, some materials exhibit further complexity by overlapping the broad domains above, such as in multiferroicity wherein two or more ferroic orders, such as ferromagnetism, ferroelectricity, or ferroelasticity, coexist within a single material. When integrated in JJs as barriers, such multiferroic systems provide additional internal degrees of freedom including unique phonon interactions, enabling coupled electric, magnetic, and structural control of the superconducting phase and current.^12,13^

Several excellent reviews have addressed specific subclasses of unconventional Josephson systems, including superconducting spintronics, ferromagnetic Josephson junctions, topological weak links, and superconducting diode effects.^7,14–18^ Rather than attempting a comprehensive survey of all unconventional Josephson platforms, this mini-review focuses specifically on barrier-driven QMJJs in which collective magnetic, correlated-electronic, or ferroelectric order within the barrier actively modifies the superconducting response. We organize the discussion around three representative barrier classes: magnetic barriers, where spin order and exchange interactions reshape superconducting coherence; correlated barriers, where many-body electronic effects modify transport symmetry and proximity behavior; and ferroelectric or multiferroic barriers, where switchable polarization enables electrically tunable superconducting transport.

Importantly, these material platforms exist at markedly different levels of experimental maturity, ranging from well-established ferromagnetic Josephson junctions to emerging altermagnetic, correlated, and ferroelectric systems where many predicted phenomena remain experimentally unresolved. Our goal is therefore not only to summarize recent progress but also to highlight conceptual connections between these material classes, clarify distinctions in their experimental status, and identify promising routes toward programmable superconducting functionality based on quantum materials. Figure 1 and Table 1 outline representative quantum-material barriers and the unconventional Josephson phenomena they enable.

**Figure 1:**
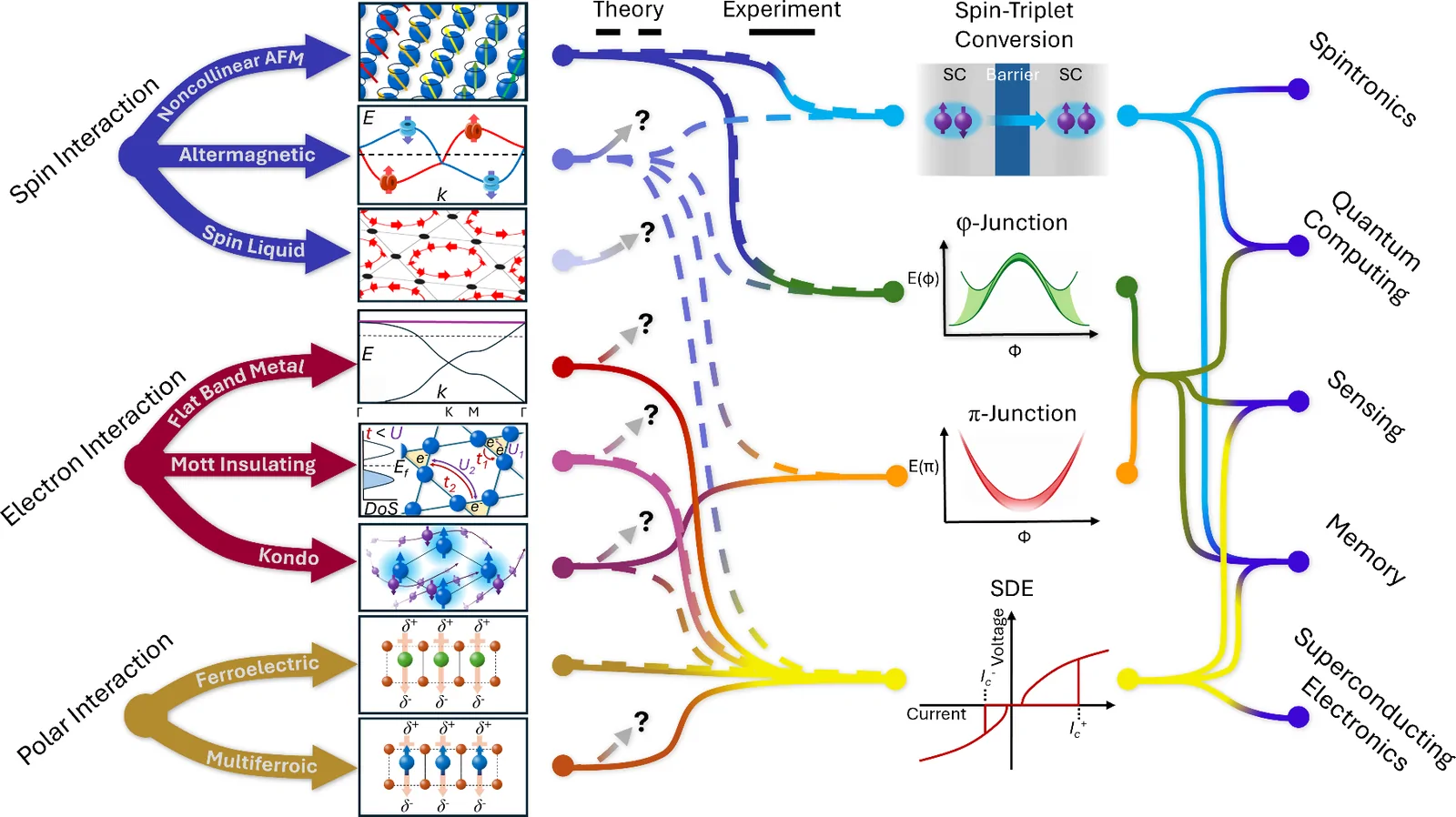
Schematic of varying unconventional barrier classes and corresponding QMJJ phenomena. Dashed and solid lines highlight their respective theory and experimental paths. Arrows with the question marks suggest areas that have yet to be investigated, highlighting literature gaps.

**Table 1:** Summary of the Barrier Classes in Quantum Material Josephson Junctions and Their Current Progress

Barrier Class	Sub Class	Key Parameter	Theoretical	Experimental	Open Questions
Spin Interacting	Noncollinear AFM^a^	Magnetic structure symmetry	Enhanced Josephson supercurrent^29^	Long-range proximity effect^30^	Do noncollinear spin systems of trivial topology host similar behavior?
	Altermagnets	Spin split electronic bands	Josephson diode effect,^36^ spin triplet conversion,^37^ 0−π transition,^37^ Majorana bound states^39^	No literature found	Does experiment confirm the predicted effects made by theory?
	Spin liquid	Strong magnetic fluctuations with no order	No literature found		How are JJs influenced by spatially disordered magnetism and spin excitations?
Electronic interacting	Flat band metal	(near) Zero band dispersion near *E*_*F*_	Long-range proximity effect^50^	Josephson diode effect and twisted bilayer graphene^50^	What microscopic mechanism mediates coupling to Cooper pairs?
	Mott insulator	Coulomb repulsion-electron kinetic energy/hopping ratio (*U*/W or *U*/*t*)	Temperature-stable supercurrent,^40^ Mott-tuned transport,^41^ proximitized Mott transition^49^	Field-free supercurrent rectification^42^ and correlation-enhanced rectification^54^	How does the Josephson critical current scale with the size of the Mott gap? What microscopic process(es) give rise to the field free superconducting diode effect?
	Kondo metal^b^	Local moment-conduction electron hybridization and exchange interaction	Kondo-induced 0−π transition,^52^ and 0′−π′ phase detection^53^	Kondo Josephson transport,^55^ and gate-tunable Kondo transport^56^	Do Kondo correlations and superconducting pairing hybridize within the barrier region?
Dipole interacting	Ferroelectric	Nonuniform, switchable internal electric field	Polarization-dependent supercurrent^65,66^	Nonvolatile memory in thin film device^67,68^	What is the mechanism that FE polarization affects critical current across the JJ?
			Memristive behavior^69^	Switchable Josephson diode effect^72^	
	Multiferroic	Coexisting nonuniform, switchable internal electric and magnetic field	No literature found	Field-free Josephson diode effect^71^	Do the coupling polar and magnet order interact to create novel control of superconducting devices?

a Here the references only reflect topologically nontrivial noncollinear AFMs, as no references for general noncollinear AFMs were found.

b Here, the references only reflect quantum dots in the Kondo regime, as references for bulk Kondo materials were found.

Ferromagnetic Josephson junctions represent the most experimentally mature and extensively established class of quantum-material barriers. Magnetic barrier materials influence superconductivity in JJs through the net exchange interaction experienced by a Cooper pair over the superconducting coherence length within the barrier (schematic of various scenarios discussed here is showcased in Figure 2). Superconducting junctions incorporating ferromagnetic barriers (S/F/S) are the most studied type of superconductor/magnetic material/superconductor (S/M/S) JJs, as their nonzero magnetization provides a platform for investigating spin-dependent transport and superconducting proximity effects. In conventional ferromagnetic junctions, the collinear exchange field lifts spin degeneracy, leading to oscillatory and rapidly decaying superconducting pair correlations and the emergence of π-junction behavior and strong suppression of conventional spin-singlet superconductivity.^14,19^ Ferromagnetic π-junction behavior was experimentally established more than two decades ago, most notably by Ryazanov et al. in Nb/CuNi/Nb junctions, where a temperature-driven sign change of the critical current provided evidence for a 0−π transition, and by Kontos et al., who observed damped oscillations of the critical current with ferromagnetic-layer thickness in SIFS junctions.^20,21^ These experiments firmly established that exchange fields in ferromagnetic barriers can reverse the Josephson ground-state phase. Since then, the field has also experimentally demonstrated controllable 0−π switching, spin-valve behavior, and long-range triplet supercurrents in engineered magnetic multilayers and half-metallic systems. However, translation into superconducting memory and logic technologies has progressed more slowly than the underlying physics might suggest. In addition to challenges associated with stray magnetic fields, flux trapping, and maintaining large characteristic voltages, the superconducting response is highly sensitive to interfacial roughness, magnetic domain configuration, and nanometer-scale variations in ferromagnetic thickness, making reproducible device behavior difficult across large arrays. As a result, despite substantial progress in superconducting spintronics and phase engineering, magnetic Josephson junctions have not yet achieved the fabrication simplicity, parameter uniformity, and circuit scalability required for widespread adoption in superconducting memory and logic architectures.

**Figure 2:**
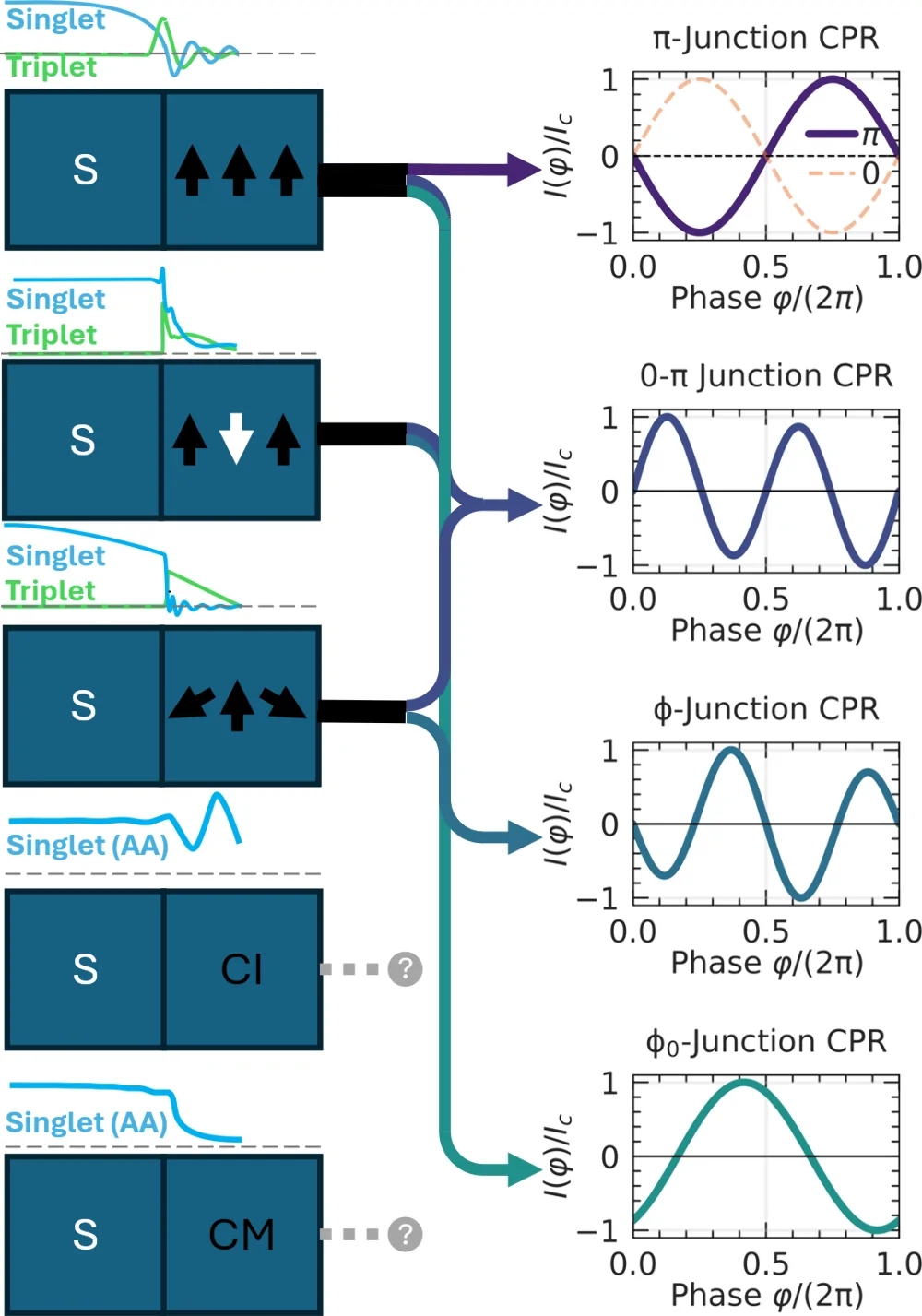
Schematic of different QMJJs, with their spatial pair amplitude and possible CPR states. Going from top to bottom, we have as a barrier a ferromagnet, an altermagnet, a noncollinear magnet, a correlated insulator (CI), and a correlated metal (CM). For these last two, the pair amplitude is approximated using the anomalous average (AA) (Freericks et al., 2001). S stands for superconductor.

As mentioned, in JJs spatially varying magnetization can transform superconducting correlations rather than simply suppress them. This process can stabilize π-junction states, generate higher-order harmonics in the current–phase relation, and produce long-range equal-spin triplet correlations.^14,19,22,23^ In most experimentally studied ferromagnetic Josephson junctions, these long-range triplet correlations are generally interpreted as odd-frequency equal-spin pairing states generated through spin mixing and magnetic noncollinearity, rather than intrinsic bulk p-wave superconductivity. Because these triplet pairs can propagate through strongly spin-polarized materials, engineered multilayer ferromagnetic junctions provide a powerful platform for studying superconductivity in complex spin environments. Additionally, when multiple spin-dependent transport channels compete, ground-state phases intermediate between 0 and π (φ-junctions) can emerge.^15,24^ In realistic junctions, these effects are often further modified by interfacial spin–orbit coupling, which can couple magnetic texture to superconducting phase accumulation and contribute to anomalous current–phase relations and nonreciprocal transport. Similar implementations have been recently investigated in antiferromagnets with varying Neel vectors.^16^

While a uniform exchange field rapidly dephases conventional spin-singlet pairs, chiral noncollinear magnetic structures can generate large fictitious fields through a nonvanishing Berry phase that allows for spin mixing and phase accumulation across the barrier, enabling conversion between singlet and triplet pairing channels. In contrast to collinear ferromagnets, these noncollinear magnets host spin structures in which the local moments are not aligned strictly parallel or antiparallel to one another. Such structures can arise from geometric frustration in lattices such as kagome or triangular systems or from relativistic interactions including Dzyaloshinskii–Moriya coupling.^25,26^ These systems often exhibit unconventional electronic responses, including anomalous Hall effects and emergent electrodynamics, even in the absence of net magnetization.^27^ More recently, kagome metals such as AV_3_Sb_5_ have attracted significant attention due to the coexistence of correlated charge order, superconductivity, and magnetic fluctuations, with evidence suggesting that noncollinear spin correlations contribute to their unusual electronic behavior.^28^

Experimental signatures of triplet-mediated supercurrents have been observed both in intrinsically noncollinear magnetic systems and in junctions where noncollinearity is engineered at interfaces. While long-range triplet transport has now been demonstrated experimentally in several engineered systems, the microscopic role of magnetic texture and interface structure remains an active area of investigation. Long-range Josephson supercurrents have been reported using the chiral noncollinear antiferromagnet Mn_3_Ge as the barrier, where triangular spin structures promote spin-triplet proximity effects without multilayer ferromagnetic engineering.^29,30^ By contrast, long-range supercurrents observed in CrO_2_, a collinear half-metallic ferromagnet, are generally interpreted as arising from spin-active interfaces rather than intrinsic magnetic noncollinearity.^8^ Similar triplet behavior has also been demonstrated in Co-based junctions incorporating weak ferromagnetic layers and in systems using Ho layers with conical spin textures acting as “triplet injectors”, both of which exhibit supercurrents persisting through thick ferromagnetic barriers.^31,32^ Noncollinear magnetic textures can additionally modify the magnetic-field dependence of the critical current *I_c_* (*B*), producing asymmetric or shifted Fraunhofer patterns that reflect the sensitivity of superconducting phase accumulation to the underlying spin structure. Magnetic noncollinearity therefore emerges as a powerful design parameter for phase batteries, programmable couplers, and other phase-sensitive superconducting elements. It should be noted that the study of noncollinear antiferromagnetic systems is concentrated toward chiral noncollinear systems due to their topological nontrivial nature. It is still unclear whether other types of noncollinear magnetic structures would also possess the properties mentioned above.

Although superconducting spin torques and supercurrent-induced magnetic dynamics have been proposed and partially explored experimentally, robust magnetization switching remains challenging because dissipationless supercurrents transfer relatively small angular momentum and require carefully engineered magnetic coupling geometries.

Frustrated magnetic systems provide a different route to spatially inhomogeneous magnetic moments. In these materials, lattice geometry or competing interactions prevent simultaneous minimization of all exchange energies, stabilizing complex spin configurations, strong fluctuations, and highly degenerate ground states.^33^ Such systems can host unconventional phases, including noncoplanar spin textures, quantum spin liquids, and spin ices, which are often accompanied by enhanced sensitivity to external perturbations such as magnetic fields, pressure, or strain.^34^ However, they have been investigated little in the context of JJs. These symmetry-rich states provide a conceptual bridge to recently identified altermagnets that exhibit spin-split electronic bands despite having zero net magnetization.

Unconventional current–phase relations and anomalous phase shifts in Josephson junctions often emerge from the combined effects of spin–orbit coupling (SOC), inversion-symmetry breaking, and noncollinear magnetism. In such systems, SOC can generate momentum-dependent spin textures that modify superconducting phase accumulation and produce anomalous current–phase relations, including finite phase offsets in the ground state (*ϕ*_0_-junction behavior). While noncollinear magnetism can independently generate spin mixing and triplet conversion, SOC provides an additional mechanism for coupling spin polarization to superconducting phase coherence, enabling nonreciprocal transport and anomalous Josephson effects even in systems without large net magnetization. Experimentally, anomalous phase shifts and *ϕ*_0_-junction behavior have already been demonstrated in spin–orbit-coupled Josephson systems, most notably in the nanowire-based junctions studied by Szombati et al., where Rashba SOC combined with Zeeman splitting generated a finite phase offset in the Josephson relation.^35^ These experiments established that inversion-symmetry breaking and SOC alone can produce unconventional phase behavior without requiring intrinsically complex magnetic order.

In this context, altermagnets represent a conceptually distinct platform for Josephson physics because their symmetry-protected spin splitting can modify superconducting phase coherence despite the absence of net magnetization. Conventional spin–orbit-coupled ϕ_0_-junctions generally rely on externally applied magnetic fields, Rashba-type inversion asymmetry, or engineered heterostructures to lift spin degeneracy. By contrast, altermagnets intrinsically host momentum-dependent spin splitting protected by crystal symmetry despite exhibiting zero net magnetization. This distinction is potentially important because it could enable spin-selective superconducting transport, anomalous phase shifts, and nonreciprocal Josephson effects without macroscopic magnetic fields or strong ferromagnetic exchange. However, unlike SOC-based ϕ_0_-junctions, which are experimentally well established, most altermagnetic Josephson predictions presently remain theoretical. Key open questions include the robustness of symmetry-protected spin splitting at realistic interfaces, the role of disorder and interfacial reconstruction, and whether altermagnetic barriers can experimentally generate phase shifts or triplet transport comparable to those already realized in spin–orbit-coupled and ferromagnetic systems.

Unlike long-range triplet transport in ferromagnetic multilayers, which has been experimentally demonstrated, to date, experimental realizations of altermagnetic Josephson junctions remain extremely limited, and many predicted effects have not yet been verified experimentally It has been predicted that altermagnets should show a superconducting diode effect dependent upon their crystallographic symmetry due to time reversal symmetry breaking.^36^ In addition to diode effects, altermagnetic barriers are predicted to enable singlet–triplet conversion, anomalous Josephson currents, and tunable 0−π transitions.^37^ Their symmetry-driven spin textures may also stabilize finite-momentum Cooper pairing analogous to Fulde–Ferrell–Larkin–Ovchinnikov states, but arising from crystal symmetry rather than Zeeman fields.^38^ Perhaps most intriguingly, altermagnetic JJs have been proposed as platforms for topological superconductivity and Majorana bound states in the absence of net magnetization. Recent theoretical work demonstrates that altermagnetic order can provide the effective spin polarization required to stabilize Majorana modes while avoiding many of the challenges associated with ferromagnetic or externally biased systems.^39^ Although spin-triplet supercurrents have already been realized in engineered ferromagnetic heterostructures, these systems often require multilayer magnetic engineering, spin-active interfaces, or finite magnetization that can introduce stray fields and pair-breaking effects. Altermagnets are theoretically appealing because they may provide symmetry-driven spin splitting within magnetically compensated systems, potentially enabling similar functionality without macroscopic magnetization. These theoretical proposals highlight altermagnets as a potentially compelling platform for future superconducting quantum devices, although experimental validation remains limited. However, this field is still emerging, and experimental validation for many of these claims remains to be seen.

Another representative class of QMJJ barriers are strongly correlated materials. Compared with magnetic Josephson junctions, correlated-barrier systems remain significantly less experimentally developed, with much of the field still driven by theoretical modeling and a comparatively small number of experimental realizations. To date, JJs incorporating strongly correlated barriers (S/CB/S) have been explored primarily at the theoretical level, with only a limited number of experimental studies reported.^40–42^ Correlated materials in this case are a broad class of materials that have flat bands close to or at the Fermi level.^43–46^ These flat bands form due to strong electron–electron interactions (through, for example, Coulomb or spin interactions, which can create Mott insulators or heavy fermion systems, respectively), such that they are of the same order or bigger than the kinetic energy of the electrons. These systems can show a multitude of emergent states, such as strange metallicity or non-BCS superconductivity. Intuitively, having Cooper pairs travel through or interact with a material in which the electron–electron interactions are strong would influence their behavior and resulting JJ properties. This was shown in early theoretical work, which modeled S/CB/S junctions as superconducting leads separated by a correlated barrier composed of several atomic layers, where correlations are introduced through on-site interactions tuned across a Mott metal–insulator transition.^40,41,47,48^ In the weakly correlated metallic regime, the junction behaves similarly to an SNS system, exhibiting nonsinusoidal CPR that depends on the barrier thickness, with the maximum supercurrent occurring at a phase different from π/2. As the correlation strength increases and the barrier approaches a Mott insulating state, the CPR evolves toward a sinusoidal form characteristic of tunneling junctions. Near the metal–insulator transition, however, strong correlation effects can lead to interface-pinned oscillations of the pair amplitude and an enhancement of the characteristic voltage *I*_*c*_ *R*_*n*_, which may exceed the Ambegaokar–Baratoff (AB) limit for specific barrier thicknesses. When the system enters the fully insulating regime, the CPR becomes sinusoidal, but the critical current is strongly suppressed relative to the AB prediction. Related dynamical Mean-Field Theory (DMFT) results have also been obtained using Hubbard-model descriptions with Bethe-lattice self-consistency.^49^

Strong electronic correlations can also generate unconventional proximity effects in flat-band metal systems. While these systems have been largely absent from the literature on JJs, some theoretical studies predict that they may exhibit enhanced superconducting proximity effects compared to conventional S/N junctions, where the de Gennes theory predicts zero proximity effect due to the absence of band dispersion.^50^ In such systems, the temperature dependence of the critical current can deviate strongly from conventional behavior and may even become nonmonotonic. Some of these effects have been observed experimentally on twisted bilayer graphene (TBG) barrier Josephson junctions.^51^ When the TBG barrier is tuned to the flat-band regime, the measured critical current is enhanced compared with expectations for a normal metallic barrier, indicating that flat-band and correlated electronic states can significantly modify the Josephson transport.

Whereas Mott and flat-band barriers primarily modify collective electronic coherence within extended systems, quantum dot Josephson junctions provide a localized strongly interacting limit in which Coulomb correlations dominate the transport physics.

Another theoretically explored realization of correlated-barrier Josephson physics is the quantum dot (QD) junction, where correlations arise through the Kondo effect. In this regime, the Josephson effect can undergo a 0−π transition when the Kondo temperature becomes comparable to the superconducting critical temperature.^52,53^ Outside the Kondo regime (Kondo temperature (*T*_k_) ≪ superconducting gap (Δ)), inversion-symmetry breaking through chirality provides an alternative route for correlations to modify the junction behavior. Here, chirality is treated as an intrinsic property of the quantum dot rather than arising from asymmetry between the superconducting leads, allowing for multiple possible microscopic realizations. In this case, electronic correlations can generate both a 0−π transition and field-free Josephson diode behavior.^54^ The magnitude of this effect depends on both the coupling strength between the superconducting leads and the QD, with a better coupling (relative correlation *U*/Δ) leading to a bigger effect. Experimentally, the 0−π transition in the Kondo regime has been seen.^55,56^ However, Josephson diode behavior in chirally coupled QD junctions has not yet been reported.

For correlated insulating barriers (S/CI/S), recent experiments on NbSe_2_/Nb_3_X_8_/NbSe_2_ junctions, where Nb_3_X_8_ (X = Cl, Br, I) is a breathing-mode kagome lattice insulator, demonstrate strong modifications of the conventional Josephson effect.^42,54^ These junctions exhibit a field-free Josephson diode effect, producing a pronounced asymmetry between the positive and negative critical currents (*I*_c+_ and *I*_c–_) even in the absence of an external magnetic field.^57^ Although these observations provide strong experimental evidence for correlation-driven nonreciprocal Josephson transport, the microscopic origin of the diode response and the relative roles of symmetry breaking, correlations, and interfacial effects remain under active debate. The diode efficiency is largest for devices incorporating the most strongly correlated barrier and disappears for weakly correlated barriers. Further work has shown that this effect can be electrostatically tuned in Nb_3_Cl_8_ devices via gating.^42^ Additionally, the temperature dependence of the critical current deviates strongly from the classical AB relation, with *I*_*c*_ significantly suppressed relative to conventional predictions.^41^ An open question still is how time reversal symmetry is broken in these systems. Even though the Nb_3_X_8_ are not ferromagnetic, their exact magnetic ground state is unknown. This makes reproducing these results with a more studied correlated insulator a priority, to understand the role of correlation versus the magnetic state.

Superconductivity in kagome correlated metals such as the AV_3_Sb_5_ family (A = K, Rb, Cs) has also attracted significant attention, with experiments suggesting intrinsic time-reversal symmetry breaking and the presence of chiral superconducting domains.^58–60^ However, because these studies probe the superconducting state of AV_3_Sb_5_ itself, it remains difficult to disentangle the microscopic origins of the observed phenomena: whether they arise from strong electronic correlations, unconventional magnetic order, or intrinsically chiral superconducting pairing. In contrast, there have been reports of employing nonstoichiometric, nonsuperconducting K_1–*x*_V_3_Sb_5_ as a barrier material in a JJ.^61^ These junctions exhibit a strongly anisotropic magnetic field response: an in-plane field shows a somewhat traditional Fraunhofer pattern, while an out-of-plane field induces rapid oscillations with a critical current minimum near zero field.^61^ This behavior is consistent with the emergence of an anisotropic internal magnetic field, together with edge currents in K_1–*x*_V_3_Sb_5_.

Collectively, these studies demonstrate that strongly correlated barriers cannot be treated as passive tunneling regions within conventional Josephson frameworks. Instead, many-body interactions within the barrier directly reshape superconducting phase coherence, transport symmetry, and the resulting current–phase relation. The now more developing theory on these junction types already shows that pure correlation, even without spin interaction, alters the behavior significantly. Experimentally disentangling pure correlation effects from possible correlation-induced magnetic properties is a hard task that will need more research. Although experimentally underexplored, correlated-barrier junctions already exhibit signatures of nonreciprocal transport, anomalous magnetic-field responses, and substantial deviations from conventional Ambegaokar–Baratoff behavior. In this regime, the barrier acts not merely as an unconventional tunneling medium but as an interacting quantum system that imprints correlation-driven electronic structure and symmetry onto the superconducting response itself. While the experimental landscape remains relatively limited, these studies suggest that correlated-barrier Josephson junctions may provide a promising platform for exploring many-body superconducting transport phenomena beyond conventional noninteracting descriptions.

Recent advances in ferroelectric growth and fabrication, together with the emergence of exfoliable van der Waals ferroelectrics and multiferroics, have enabled new opportunities to explore superconducting transport at ferroelectric–superconductor interfaces, including the use of ferroelectric materials as barriers in Josephson junctions.^18,62–64^ Unlike magnetic and correlated barriers, which primarily modify superconducting phase coherence through spin or many-body electronic interactions, ferroelectric barriers provide electrically switchable control over the tunneling potential itself. Although this field remains at an early stage, both theoretical and experimental studies suggest that ferroelectric order can provide a versatile route for tuning Josephson transport. At present, however, most ferroelectric Josephson platforms remain proof-of-concept demonstrations rather than technologically mature device architectures.

Building on the established framework of ferroelectric tunnel junctions (FTJs), theoretical studies predict that asymmetric ferroelectric Josephson junctions can exhibit polarization-dependent critical currents, producing electrically tunable supercurrents as illustrated in Figure 3a.^65,66^ Such asymmetry may be engineered through barrier design, for example, by incorporating dielectric layers of unequal thickness. At ferroelectric–superconductor interfaces, polarization-induced surface charges can additionally modify superconducting properties including the critical temperature and critical current, providing a nonvolatile control mechanism that has been exploited in cryogenic memory devices.^63,67,68^ Beyond static control of transport, coupling between the Josephson current and ferroelectric polarization can also generate nonlinear dynamics. Early studies predicted oscillatory polarization behavior, while more recent work based on resistively and capacitively shunted junction (RCSJ) models suggests the possibility of memristive superconducting behavior and ferroelectric Josephson memristors.^69^

**Figure 3:**
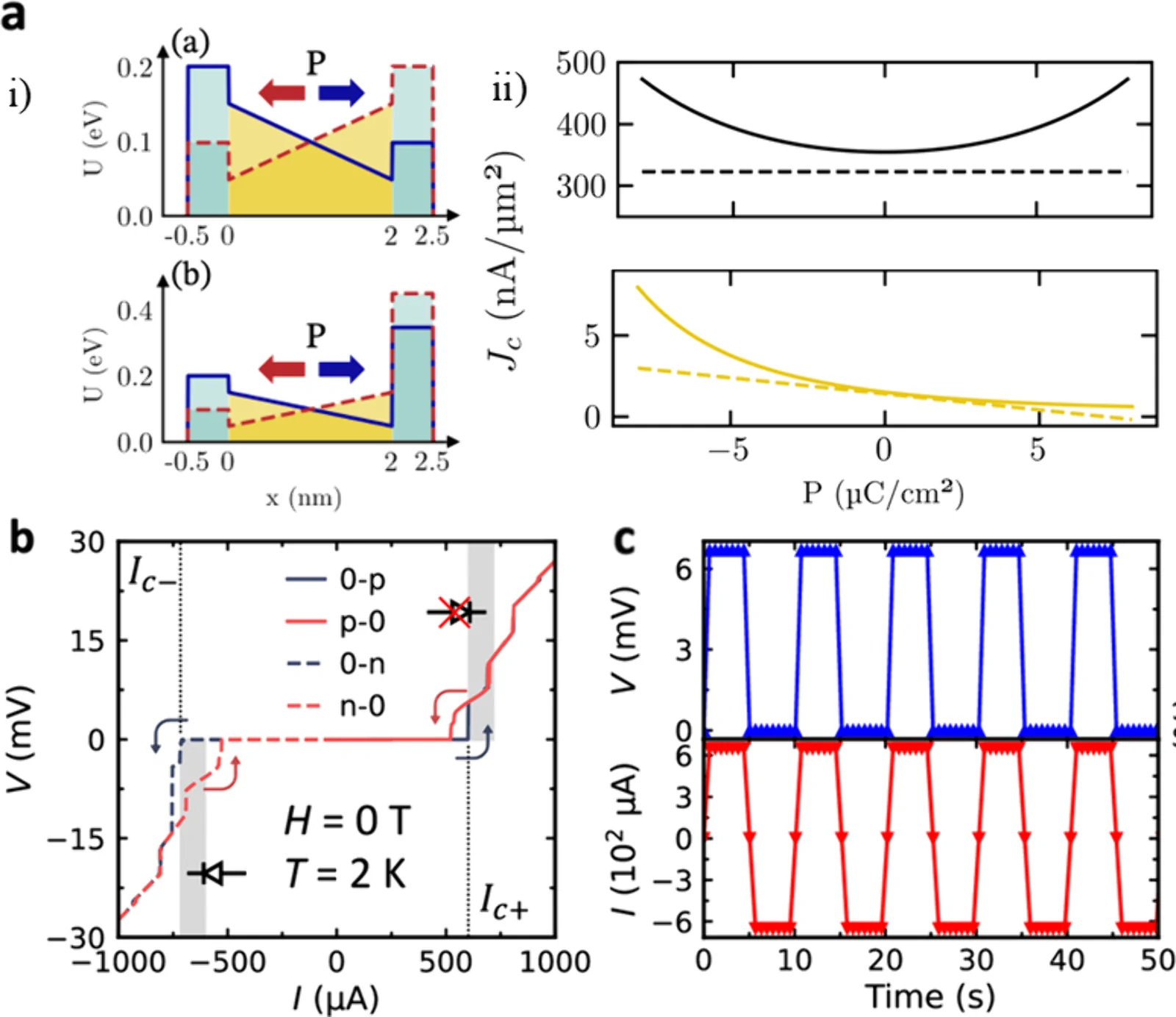
(a) Polarization-controlled supercurrent in a ferroelectric Josephson junction. (i) Schematic potential profiles for symmetric (top) and asymmetric (bottom) dielectric–ferroelectric–dielectric barriers. Blue (solid) and red (dashed) curves represent opposite polarization directions. (ii) Calculated critical current density as a function of ferroelectric polarization for symmetric and asymmetric barriers (solid: numerical calculation; dashed: analytical approximation).^66,71^ (b) *V*–*I* characteristics of the NiI_2_ junction. Current sweep directions (0–p, p–0, 0–n, n–0) correspond to 0 → +1000 μA, +1000 → 0 μA, 0 → −1000 μA, and −1000 → 0 μA. The critical currents *I*_*c*_^+600 μA^ and *I*_*c*_^–=718 μA^ are defined by the first switching events in the 0–p and 0–n curves. The shaded region marks the diode operating range. (c) Demonstration of supercurrent rectification of the NiI_2_ junction *I*_bias_ = ±650 μA.^67,70–73^

Experimentally, the first demonstrations of ferroelectric/multiferroic barriers into JJs and ferroelectric/superconductor interface devices have already been reported (Figure 3b,c).^70^ These systems exhibit electrically tunable supercurrents, superconducting diode behavior, and electrostatically controlled transport, although the microscopic mechanisms governing many of these effects remain under active investigation. The combination of superconductivity with switchable ferroelectric order opens possible pathways toward superconducting diodes, field-effect devices, and cryogenic memory architectures. Additional control strategies, including terahertz excitation, optical manipulation of ferroelectric order, and magnetic tuning in multiferroic junctions, may further expand the functionality of these hybrid systems.

## Conclusion

This mini-review summarized how unconventional magnetic order, electronic correlations, and polarization can modify Josephson transport beyond common tunnel-junction behavior. Across ferromagnetic, noncollinear, altermagnetic, correlated, and ferroelectric barriers, the superconducting current–phase relation reflects the underlying spin, correlation, and polarization landscape of the barrier material. Recent experiments demonstrating phase shifts, higher-harmonic contributions, and nonreciprocal Josephson effects show that these junctions can access regimes of superconducting transport beyond those described by standard proximity-effect models.

At the same time, many of the phenomena discussed throughout this review remain experimentally unresolved or only partially understood. Although ferromagnetic Josephson junctions are now a mature platform for studying π-junction behavior and spin-triplet transport, important challenges remain in achieving scalable device reproducibility, minimizing interfacial disorder, and controlling magnetic inhomogeneity at technologically relevant dimensions. For altermagnetic Josephson systems, the central unresolved question is whether symmetry-protected spin splitting can robustly survive realistic interfaces and produce experimentally measurable superconducting phase effects distinct from those already realized in spin–orbit-coupled or ferromagnetic heterostructures. Similarly, in correlated and kagome-barrier junctions, the microscopic origin of field-free Josephson diode behavior and anomalous current–phase relations remain under active debate, particularly regarding the relative roles of electronic correlations, symmetry breaking, and interfacial reconstruction. Ferroelectric Josephson junctions additionally face open questions concerning long-term polarization stability, switching reproducibility at cryogenic temperatures, and the extent to which polarization-driven superconducting control can be integrated into scalable superconducting circuitry.

Addressing these challenges will require advances not only in materials synthesis and interface engineering but also in phase-sensitive and spatially resolved experimental probes capable of disentangling magnetic, correlated, and ferroic contributions to Josephson transport. Experiments combining tunneling spectroscopy, microwave measurements, spin-sensitive probes, and electrostatic control will likely be particularly important for distinguishing intrinsic barrier-driven phenomena from interfacial or nonequilibrium effects. More broadly, future progress in quantum material Josephson junctions will depend on establishing clearer connections between microscopic barrier physics and reproducible device functionality. If these challenges can be overcome, then Josephson junctions incorporating quantum materials may ultimately provide experimentally tunable platforms for unconventional superconductivity, superconducting spintronics, and electrically programmable quantum devices.
